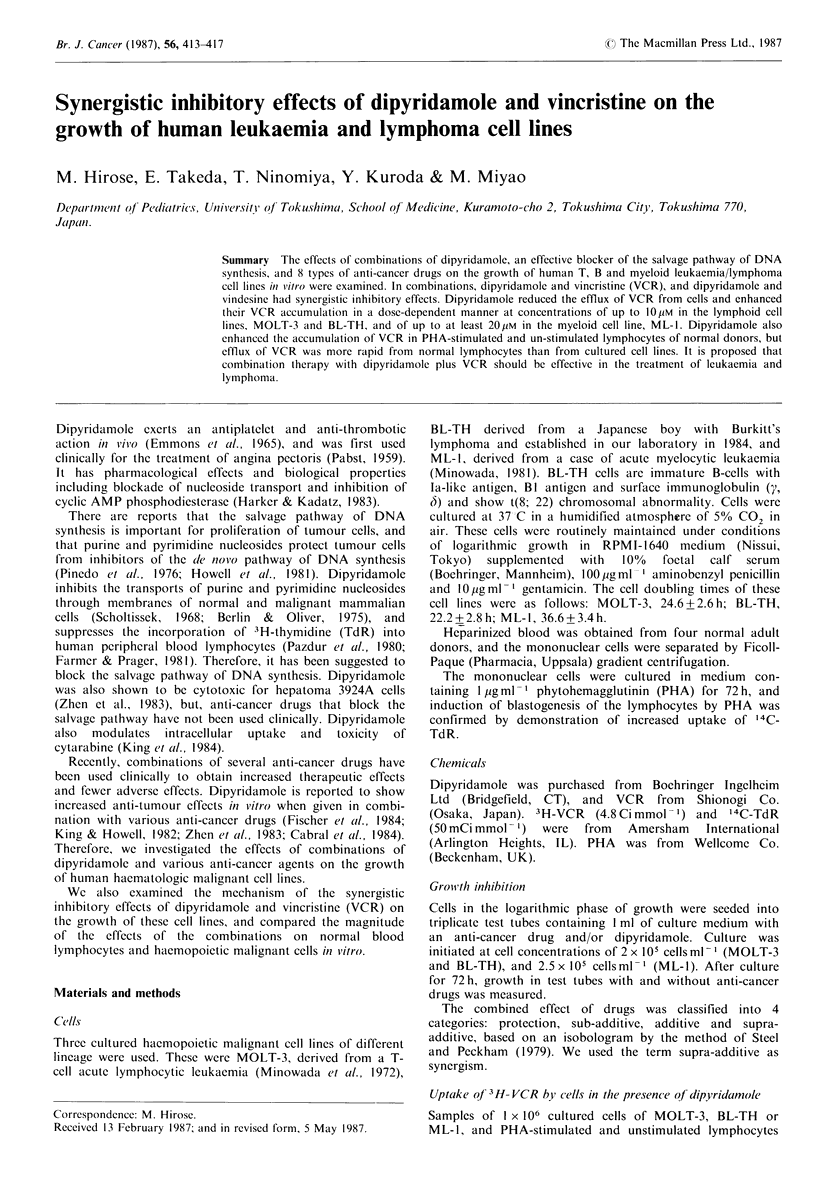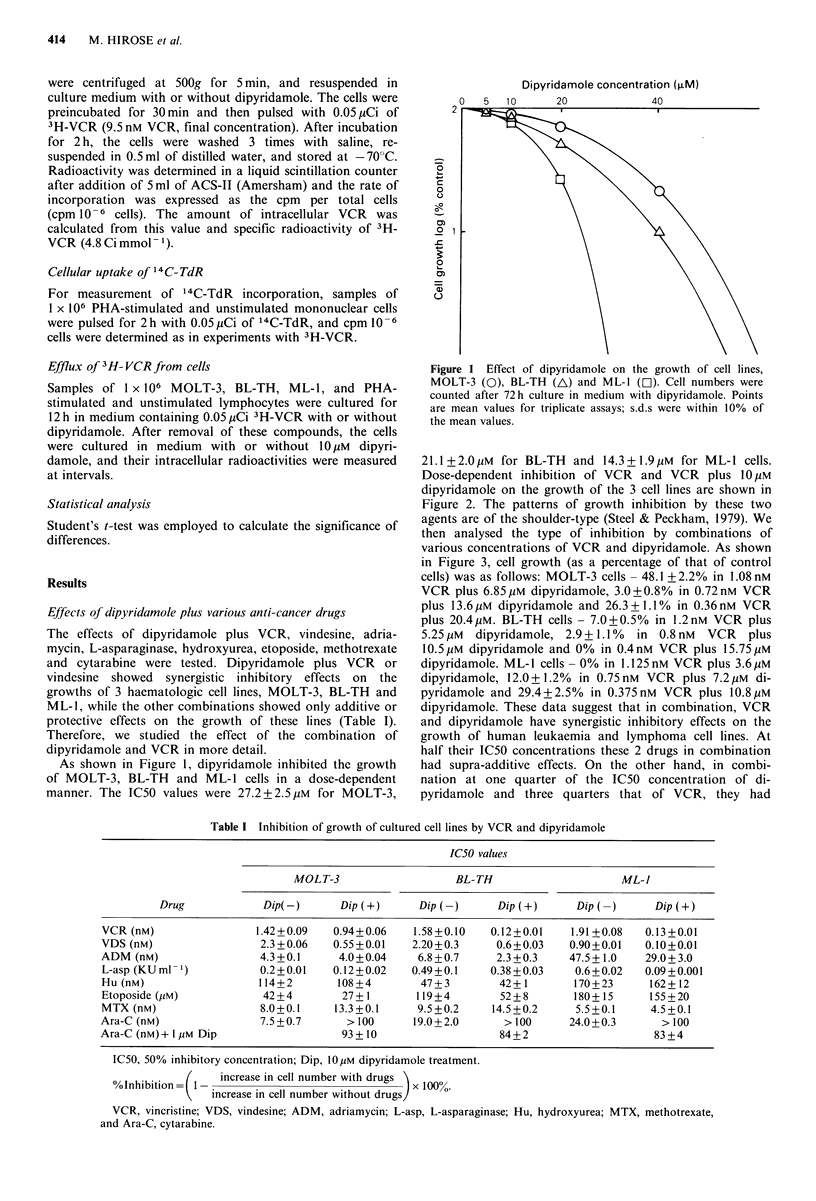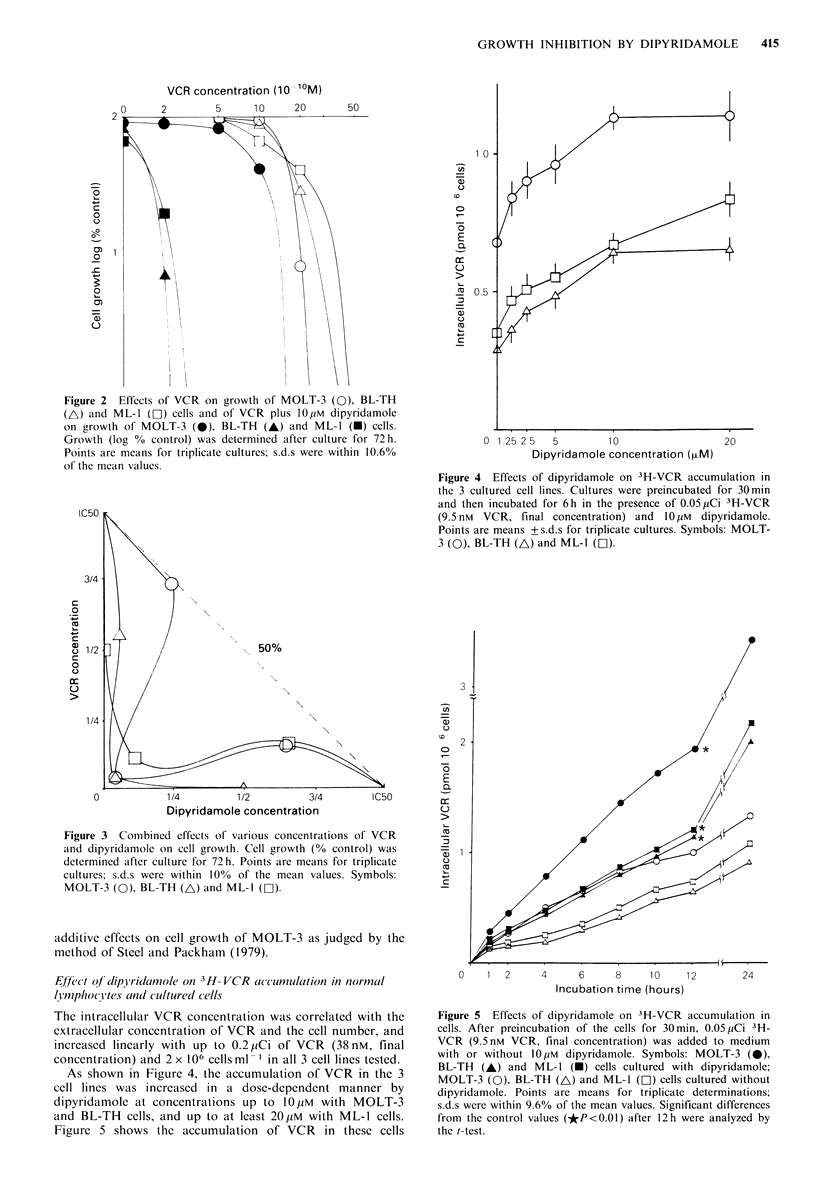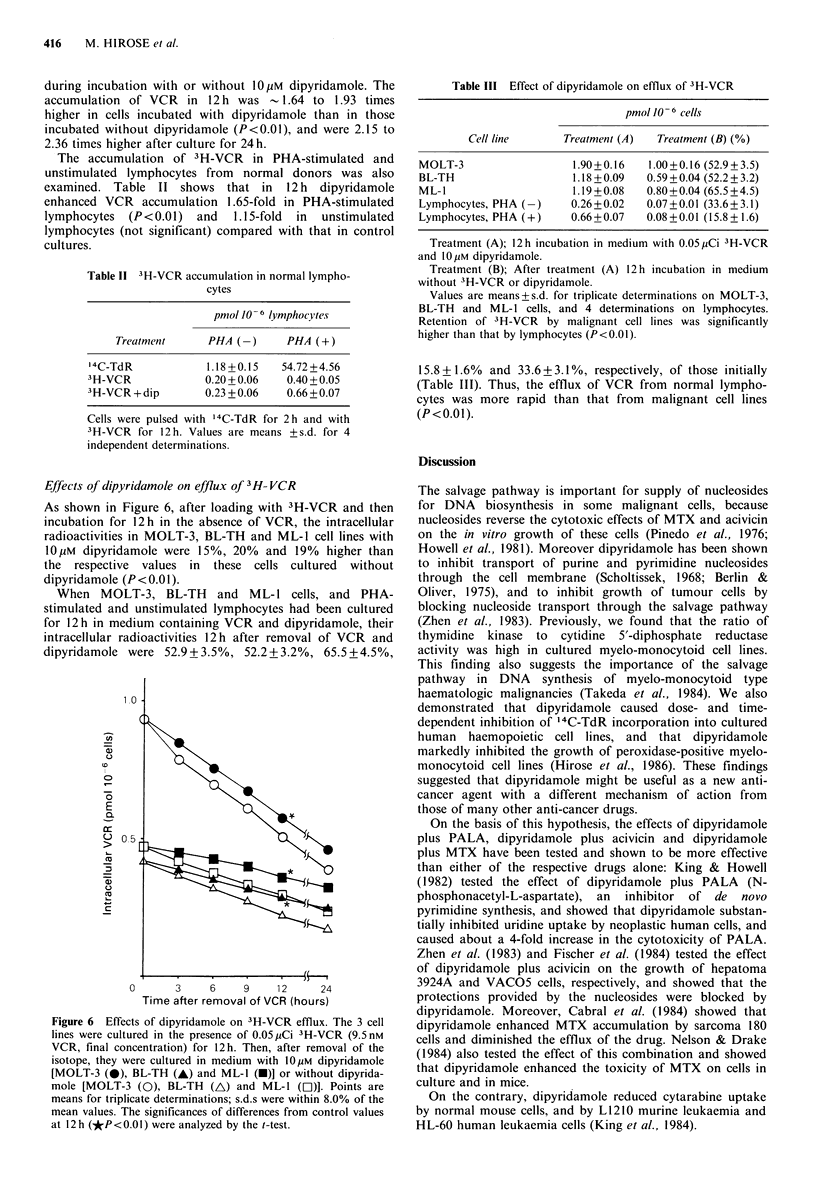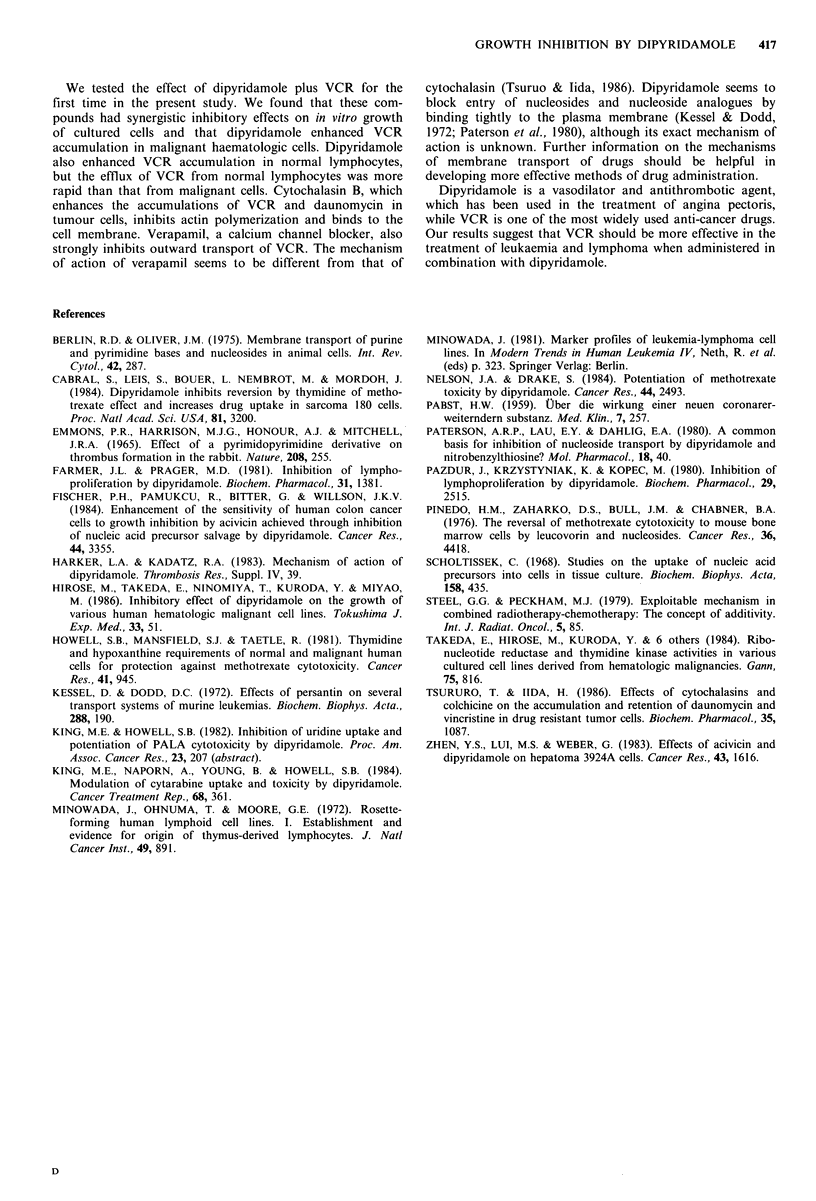# Synergistic inhibitory effects of dipyridamole and vincristine on the growth of human leukaemia and lymphoma cell lines.

**DOI:** 10.1038/bjc.1987.216

**Published:** 1987-10

**Authors:** M. Hirose, E. Takeda, T. Ninomiya, Y. Kuroda, M. Miyao

**Affiliations:** Department of Pediatrics, University of Tokushima, School of Medicine, Japan.

## Abstract

The effects of combinations of dipyridamole, an effective blocker of the salvage pathway of DNA synthesis, and 8 types of anti-cancer drugs on the growth of human T, B and myeloid leukaemia/lymphoma cell lines in vitro were examined. In combinations, dipyridamole and vincristine (VCR), and dipyridamole and vindesine had synergistic inhibitory effects. Dipyridamole reduced the efflux of VCR from cells and enhanced their VCR accumulation in a dose-dependent manner at concentrations of up to 10 microM in the lymphoid cell lines, MOLT-3 and BL-TH, and of up to at least 20 microM in the myeloid cell line, ML-1. Dipyridamole also enhanced the accumulation of VCR in PHA-stimulated and un-stimulated lymphocytes of normal donors, but efflux of VCR was more rapid from normal lymphocytes than from cultured cell lines. It is proposed that combination therapy with dipyridamole plus VCR should be effective in the treatment of leukaemia and lymphoma.


Br. J. Cancer (1987), 56, 413–417 © The Macmillan Press Ltd., 1987

### Synergistic inhibitory effects of dipyridamole and vincristine on the growth of human leukaemia and lymphoma cell lines

M. Hirose, E. Takeda, T. Ninomiya, Y. Kuroda & M. Miyao

*Department of Pediatrics, University of Tokushima, School of Medicine, Kuramoto-cho 2, Tokushima City, Tokushima 770, Japan.*

**Summary** The effects of combinations of dipyridamole, an effective blocker of the salvage pathway of DNA synthesis, and 8 types of anti-cancer drugs on the growth of human T, B and myeloid leukaemia/lymphoma cell lines *in vitro* were examined. In combinations, dipyridamole and vincristine (VCR), and dipyridamole and vindesine had synergistic inhibitory effects. Dipyridamole reduced the efflux of VCR from cells and enhanced their VCR accumulation in a dose-dependent manner at concentrations of up to 10 $\mu$M in the lymphoid cell lines, MOLT-3 and BL-TH, and of up to at least 20 $\mu$M in the myeloid cell line, ML-1. Dipyridamole also enhanced the accumulation of VCR in PHA-stimulated and un-stimulated lymphocytes of normal donors, but efflux of VCR was more rapid from normal lymphocytes than from cultured cell lines. It is proposed that combination therapy with dipyridamole plus VCR should be effective in the treatment of leukaemia and lymphoma.

Dipyridamole exerts an antiplatelet and anti-thrombotic action *in vivo* (Emmons *et al.*, 1965), and was first used clinically for the treatment of angina pectoris (Pabst, 1959). It has pharmacological effects and biological properties including blockade of nucleoside transport and inhibition of cyclic AMP phosphodiesterase (Harker & Kadatz, 1983).

There are reports that the salvage pathway of DNA synthesis is important for proliferation of tumour cells, and that purine and pyrimidine nucleosides protect tumour cells from inhibitors of the *de novo* pathway of DNA synthesis (Pinedo *et al.*, 1976; Howell *et al.*, 1981). Dipyridamole inhibits the transports of purine and pyrimidine nucleosides through membranes of normal and malignant mammalian cells (Scholtissek, 1968; Berlin & Oliver, 1975), and suppresses the incorporation of $^3$H-thymidine (TdR) into human peripheral blood lymphocytes (Pazdur *et al.*, 1980; Farmer & Prager, 1981). Therefore, it has been suggested to block the salvage pathway of DNA synthesis. Dipyridamole was also shown to be cytotoxic for hepatoma 3924A cells (Zhen et al., 1983), but, anti-cancer drugs that block the salvage pathway have not been used clinically. Dipyridamole also modulates intracellular uptake and toxicity of cytarabine (King *et al.*, 1984).

Recently, combinations of several anti-cancer drugs have been used clinically to obtain increased therapeutic effects and fewer adverse effects. Dipyridamole is reported to show increased anti-tumour effects *in vitro* when given in combination with various anti-cancer drugs (Fischer *et al.*, 1984; King & Howell, 1982; Zhen *et al.*, 1983; Cabral *et al.*, 1984). Therefore, we investigated the effects of combinations of dipyridamole and various anti-cancer agents on the growth of human haematologic malignant cell lines.

We also examined the mechanism of the synergistic inhibitory effects of dipyridamole and vincristine (VCR) on the growth of these cell lines, and compared the magnitude of the effects of the combinations on normal blood lymphocytes and haemopoietic malignant cells *in vitro*.

### Materials and methods

### *Cells*

Three cultured haemopoietic malignant cell lines of different lineage were used. These were MOLT-3, derived from a T-cell acute lymphocytic leukaemia (Minowada *et al.*, 1972), BL-TH derived from a Japanese boy with Burkitt's lymphoma and established in our laboratory in 1984, and ML-1, derived from a case of acute myelocytic leukaemia (Minowada, 1981). BL-TH cells are immature B-cells with Ia-like antigen, B1 antigen and surface immunoglobulin ($\gamma$, $\delta$) and show t(8; 22) chromosomal abnormality. Cells were cultured at 37°C in a humidified atmosphere of 5% $CO_2$ in air. These cells were routinely maintained under conditions of logarithmic growth in RPMI-1640 medium (Nissui, Tokyo) supplemented with 10% foetal calf serum (Boehringer, Mannheim), 100 $\mu$g ml$^{-1}$ aminobenzyl penicillin and 10 $\mu$g ml$^{-1}$ gentamicin. The cell doubling times of these cell lines were as follows: MOLT-3, 24.6 $\pm$ 2.6 h; BL-TH, 22.2 $\pm$ 2.8 h; ML-1, 36.6 $\pm$ 3.4 h.

Heparinized blood was obtained from four normal adult donors, and the mononuclear cells were separated by Ficoll-Paque (Pharmacia, Uppsala) gradient centrifugation.

The mononuclear cells were cultured in medium containing 1 $\mu$g ml$^{-1}$ phytohemagglutinin (PHA) for 72 h, and induction of blastogenesis of the lymphocytes by PHA was confirmed by demonstration of increased uptake of $^{14}$C-TdR.

### *Chemicals*

Dipyridamole was purchased from Boehringer Ingelheim Ltd (Bridgefield, CT), and VCR from Shionogi Co. (Osaka, Japan). $^3$H-VCR (4.8 Ci mmol$^{-1}$) and $^{14}$C-TdR (50 mCi mmol$^{-1}$) were from Amersham International (Arlington Heights, IL). PHA was from Wellcome Co. (Beckenham, UK).

### *Growth inhibition*

Cells in the logarithmic phase of growth were seeded into triplicate test tubes containing 1 ml of culture medium with an anti-cancer drug and/or dipyridamole. Culture was initiated at cell concentrations of $2 \times 10^5$ cells ml$^{-1}$ (MOLT-3 and BL-TH), and $2.5 \times 10^5$ cells ml$^{-1}$ (ML-1). After culture for 72 h, growth in test tubes with and without anti-cancer drugs was measured.

The combined effect of drugs was classified into 4 categories: protection, sub-additive, additive and supra-additive, based on an isobologram by the method of Steel and Peckham (1979). We used the term supra-additive as synergism.

### *Uptake of $^3$H-VCR by cells in the presence of dipyridamole*

Samples of $1 \times 10^6$ cultured cells of MOLT-3, BL-TH or ML-1, and PHA-stimulated and unstimulated lymphocytes

Correspondence: M. Hirose.
Received 13 February 1987; and in revised form, 5 May 1987.

were centrifuged at 500g for 5 min, and resuspended in culture medium with or without dipyridamole. The cells were preincubated for 30 min and then pulsed with 0.05 $\mu$Ci of $^3$H-VCR (9.5 nM VCR, final concentration). After incubation for 2 h, the cells were washed 3 times with saline, resuspended in 0.5 ml of distilled water, and stored at $-70$°C. Radioactivity was determined in a liquid scintillation counter after addition of 5 ml of ACS-II (Amersham) and the rate of incorporation was expressed as the cpm per total cells (cpm $10^{-6}$ cells). The amount of intracellular VCR was calculated from this value and specific radioactivity of $^3$H-VCR (4.8 Ci mmol$^{-1}$).

*Cellular uptake of $^{14}$C-TdR*

For measurement of $^{14}$C-TdR incorporation, samples of $1 \times 10^6$ PHA-stimulated and unstimulated mononuclear cells were pulsed for 2 h with 0.05 $\mu$Ci of $^{14}$C-TdR, and cpm $10^{-6}$ cells were determined as in experiments with $^3$H-VCR.

*Efflux of $^3$H-VCR from cells*

Samples of $1 \times 10^6$ MOLT-3, BL-TH, ML-1, and PHA-stimulated and unstimulated lymphocytes were cultured for 12 h in medium containing 0.05 $\mu$Ci $^3$H-VCR with or without dipyridamole. After removal of these compounds, the cells were cultured in medium with or without 10 $\mu$M dipyridamole, and their intracellular radioactivities were measured at intervals.

*Statistical analysis*

Student's $t$-test was employed to calculate the significance of differences.

### Results

*Effects of dipyridamole plus various anti-cancer drugs*

The effects of dipyridamole plus VCR, vindesine, adriamycin, L-asparaginase, hydroxyurea, etoposide, methotrexate and cytarabine were tested. Dipyridamole plus VCR or vindesine showed synergistic inhibitory effects on the growths of 3 haematologic cell lines, MOLT-3, BL-TH and ML-1, while the other combinations showed only additive or protective effects on the growth of these lines (Table I). Therefore, we studied the effect of the combination of dipyridamole and VCR in more detail.

As shown in Figure 1, dipyridamole inhibited the growth of MOLT-3, BL-TH and ML-1 cells in a dose-dependent manner. The IC50 values were 27.2 ± 2.5 $\mu$M for MOLT-3, 21.1 ± 2.0 $\mu$M for BL-TH and 14.3 ± 1.9 $\mu$M for ML-1 cells. Dose-dependent inhibition of VCR and VCR plus 10 $\mu$M dipyridamole on the growth of the 3 cell lines are shown in Figure 2. The patterns of growth inhibition by these two agents are of the shoulder-type (Steel & Peckham, 1979). We then analysed the type of inhibition by combinations of various concentrations of VCR and dipyridamole. As shown in Figure 3, cell growth (as a percentage of that of control cells) was as follows: MOLT-3 cells – 48.1 ± 2.2% in 1.08 nM VCR plus 6.85 $\mu$M dipyridamole, 3.0 ± 0.8% in 0.72 nM VCR plus 13.6 $\mu$M dipyridamole and 26.3 ± 1.1% in 0.36 nM VCR plus 20.4 $\mu$M. BL-TH cells – 7.0 ± 0.5% in 1.2 nM VCR plus 5.25 $\mu$M dipyridamole, 2.9 ± 1.1% in 0.8 nM VCR plus 10.5 $\mu$M dipyridamole and 0% in 0.4 nM VCR plus 15.75 $\mu$M dipyridamole. ML-1 cells – 0% in 1.125 nM VCR plus 3.6 $\mu$M dipyridamole, 12.0 ± 1.2% in 0.75 nM VCR plus 7.2 $\mu$M dipyridamole and 29.4 ± 2.5% in 0.375 nM VCR plus 10.8 $\mu$M dipyridamole. These data suggest that in combination, VCR and dipyridamole have synergistic inhibitory effects on the growth of human leukaemia and lymphoma cell lines. At half their IC50 concentrations these 2 drugs in combination had supra-additive effects. On the other hand, in combination at one quarter of the IC50 concentration of dipyridamole and three quarters that of VCR, they had

**Figure 1** Effect of dipyridamole on the growth of cell lines, MOLT-3 (○), BL-TH (△) and ML-1 (□). Cell numbers were counted after 72 h culture in medium with dipyridamole. Points are mean values for triplicate assays; s.d.s were within 10% of the mean values.

**Table I** Inhibition of growth of cultured cell lines by VCR and dipyridamole

| Drug | *IC50 values* | | | | | |
|---|---|---|---|---|---|---|
| | *MOLT-3* | | *BL-TH* | | *ML-1* | |
| | *Dip(−)* | *Dip (+)* | *Dip (−)* | *Dip (+)* | *Dip (−)* | *Dip (+)* |
| VCR (nM) | 1.42 ± 0.09 | 0.94 ± 0.06 | 1.58 ± 0.10 | 0.12 ± 0.01 | 1.91 ± 0.08 | 0.13 ± 0.01 |
| VDS (nM) | 2.3 ± 0.06 | 0.55 ± 0.01 | 2.20 ± 0.3 | 0.6 ± 0.03 | 0.90 ± 0.01 | 0.10 ± 0.01 |
| ADM (nM) | 4.3 ± 0.1 | 4.0 ± 0.04 | 6.8 ± 0.7 | 2.3 ± 0.3 | 47.5 ± 1.0 | 29.0 ± 3.0 |
| L-asp (KU ml$^{-1}$) | 0.2 ± 0.01 | 0.12 ± 0.02 | 0.49 ± 0.1 | 0.38 ± 0.03 | 0.6 ± 0.02 | 0.09 ± 0.001 |
| Hu (nM) | 114 ± 2 | 108 ± 4 | 47 ± 3 | 42 ± 1 | 170 ± 23 | 162 ± 12 |
| Etoposide ($\mu$M) | 42 ± 4 | 27 ± 1 | 119 ± 4 | 52 ± 8 | 180 ± 15 | 155 ± 20 |
| MTX (nM) | 8.0 ± 0.1 | 13.3 ± 0.1 | 9.5 ± 0.2 | 14.5 ± 0.2 | 5.5 ± 0.1 | 4.5 ± 0.1 |
| Ara-C (nM) | 7.5 ± 0.7 | > 100 | 19.0 ± 2.0 | > 100 | 24.0 ± 0.3 | > 100 |
| Ara-C (nM) + 1 $\mu$M Dip | | 93 ± 10 | | 84 ± 2 | | 83 ± 4 |

IC50, 50% inhibitory concentration; Dip, 10 $\mu$M dipyridamole treatment.

$$\%\text{Inhibition} = \left(1 - \frac{\text{increase in cell number with drugs}}{\text{increase in cell number without drugs}}\right) \times 100\%.$$

VCR, vincristine; VDS, vindesine; ADM, adriamycin; L-asp, L-asparaginase; Hu, hydroxyurea; MTX, methotrexate, and Ara-C, cytarabine.

Figure 2 Effects of VCR on growth of MOLT-3 (○), BL-TH (△) and ML-1 (□) cells and of VCR plus 10 μM dipyridamole on growth of MOLT-3 (●), BL-TH (▲) and ML-1 (■) cells. Growth (log % control) was determined after culture for 72 h. Points are means for triplicate cultures; s.d.s were within 10.6% of the mean values.

Figure 3 Combined effects of various concentrations of VCR and dipyridamole on cell growth. Cell growth (% control) was determined after culture for 72 h. Points are means for triplicate cultures; s.d.s were within 10% of the mean values. Symbols: MOLT-3 (○), BL-TH (△) and ML-1 (□).

additive effects on cell growth of MOLT-3 as judged by the method of Steel and Packham (1979).

*Effect of dipyridamole on $^3$H-VCR accumulation in normal lymphocytes and cultured cells*

The intracellular VCR concentration was correlated with the extracellular concentration of VCR and the cell number, and increased linearly with up to 0.2 μCi of VCR (38 nM, final concentration) and $2 \times 10^6$ cells ml$^{-1}$ in all 3 cell lines tested.

As shown in Figure 4, the accumulation of VCR in the 3 cell lines was increased in a dose-dependent manner by dipyridamole at concentrations up to 10 μM with MOLT-3 and BL-TH cells, and up to at least 20 μM with ML-1 cells. Figure 5 shows the accumulation of VCR in these cells

Figure 4 Effects of dipyridamole on $^3$H-VCR accumulation in the 3 cultured cell lines. Cultures were preincubated for 30 min and then incubated for 6 h in the presence of 0.05 μCi $^3$H-VCR (9.5 nM VCR, final concentration) and 10 μM dipyridamole. Points are means ± s.d.s for triplicate cultures. Symbols: MOLT-3 (○), BL-TH (△) and ML-1 (□).

Figure 5 Effects of dipyridamole on $^3$H-VCR accumulation in cells. After preincubation of the cells for 30 min, 0.05 μCi $^3$H-VCR (9.5 nM VCR, final concentration) was added to medium with or without 10 μM dipyridamole. Symbols: MOLT-3 (●), BL-TH (▲) and ML-1 (■) cells cultured with dipyridamole; MOLT-3 (○), BL-TH (△) and ML-1 (□) cells cultured without dipyridamole. Points are means for triplicate determinations; s.d.s were within 9.6% of the mean values. Significant differences from the control values (★$P < 0.01$) after 12 h were analyzed by the $t$-test.

during incubation with or without 10 μM dipyridamole. The accumulation of VCR in 12 h was ~1.64 to 1.93 times higher in cells incubated with dipyridamole than in those incubated without dipyridamole ($P<0.01$), and were 2.15 to 2.36 times higher after culture for 24 h.

The accumulation of $^{3}$H-VCR in PHA-stimulated and unstimulated lymphocytes from normal donors was also examined. Table II shows that in 12 h dipyridamole enhanced VCR accumulation 1.65-fold in PHA-stimulated lymphocytes ($P<0.01$) and 1.15-fold in unstimulated lymphocytes (not significant) compared with that in control cultures.

**Table II** $^{3}$H-VCR accumulation in normal lymphocytes

| | *pmol $10^{-6}$ lymphocytes* | |
|---|---|---|
| *Treatment* | *PHA* (−) | *PHA* (+) |
| $^{14}$C-TdR | 1.18±0.15 | 54.72±4.56 |
| $^{3}$H-VCR | 0.20±0.06 | 0.40±0.05 |
| $^{3}$H-VCR+dip | 0.23±0.06 | 0.66±0.07 |

Cells were pulsed with $^{14}$C-TdR for 2 h and with $^{3}$H-VCR for 12 h. Values are means ±s.d. for 4 independent determinations.

*Effects of dipyridamole on efflux of $^{3}$H-VCR*

As shown in Figure 6, after loading with $^{3}$H-VCR and then incubation for 12 h in the absence of VCR, the intracellular radioactivities in MOLT-3, BL-TH and ML-1 cell lines with 10 μM dipyridamole were 15%, 20% and 19% higher than the respective values in these cells cultured without dipyridamole ($P<0.01$).

When MOLT-3, BL-TH and ML-1 cells, and PHA-stimulated and unstimulated lymphocytes had been cultured for 12 h in medium containing VCR and dipyridamole, their intracellular radioactivities 12 h after removal of VCR and dipyridamole were 52.9±3.5%, 52.2±3.2%, 65.5±4.5%,

**Figure 6** Effects of dipyridamole on $^{3}$H-VCR efflux. The 3 cell lines were cultured in the presence of 0.05 μCi $^{3}$H-VCR (9.5 nM VCR, final concentration) for 12 h. Then, after removal of the isotope, they were cultured in medium with 10 μM dipyridamole [MOLT-3 (●), BL-TH (▲) and ML-1 (■)] or without dipyridamole [MOLT-3 (○), BL-TH (△) and ML-1 (□)]. Points are means for triplicate determinations; s.d.s were within 8.0% of the mean values. The significances of differences from control values at 12 h (★$P<0.01$) were analyzed by the *t*-test.

**Table III** Effect of dipyridamole on efflux of $^{3}$H-VCR

| | *pmol $10^{-6}$ cells* | |
|---|---|---|
| *Cell line* | *Treatment* (*A*) | *Treatment* (*B*) (%) |
| MOLT-3 | 1.90±0.16 | 1.00±0.16 (52.9±3.5) |
| BL-TH | 1.18±0.09 | 0.59±0.04 (52.2±3.2) |
| ML-1 | 1.19±0.08 | 0.80±0.04 (65.5±4.5) |
| Lymphocytes, PHA (−) | 0.26±0.02 | 0.07±0.01 (33.6±3.1) |
| Lymphocytes, PHA (+) | 0.66±0.07 | 0.08±0.01 (15.8±1.6) |

Treatment (A); 12 h incubation in medium with 0.05 μCi $^{3}$H-VCR and 10 μM dipyridamole.

Treatment (B); After treatment (A) 12 h incubation in medium without $^{3}$H-VCR or dipyridamole.

Values are means±s.d. for triplicate determinations on MOLT-3, BL-TH and ML-1 cells, and 4 determinations on lymphocytes. Retention of $^{3}$H-VCR by malignant cell lines was significantly higher than that by lymphocytes ($P<0.01$).

15.8±1.6% and 33.6±3.1%, respectively, of those initially (Table III). Thus, the efflux of VCR from normal lymphocytes was more rapid than that from malignant cell lines ($P<0.01$).

### Discussion

The salvage pathway is important for supply of nucleosides for DNA biosynthesis in some malignant cells, because nucleosides reverse the cytotoxic effects of MTX and acivicin on the *in vitro* growth of these cells (Pinedo *et al.*, 1976; Howell *et al.*, 1981). Moreover dipyridamole has been shown to inhibit transport of purine and pyrimidine nucleosides through the cell membrane (Scholtissek, 1968; Berlin & Oliver, 1975), and to inhibit growth of tumour cells by blocking nucleoside transport through the salvage pathway (Zhen *et al.*, 1983). Previously, we found that the ratio of thymidine kinase to cytidine 5′-diphosphate reductase activity was high in cultured myelo-monocytoid cell lines. This finding also suggests the importance of the salvage pathway in DNA synthesis of myelo-monocytoid type haematologic malignancies (Takeda *et al.*, 1984). We also demonstrated that dipyridamole caused dose- and time-dependent inhibition of $^{14}$C-TdR incorporation into cultured human haemopoietic cell lines, and that dipyridamole markedly inhibited the growth of peroxidase-positive myelo-monocytoid cell lines (Hirose *et al.*, 1986). These findings suggested that dipyridamole might be useful as a new anti-cancer agent with a different mechanism of action from those of many other anti-cancer drugs.

On the basis of this hypothesis, the effects of dipyridamole plus PALA, dipyridamole plus acivicin and dipyridamole plus MTX have been tested and shown to be more effective than either of the respective drugs alone: King & Howell (1982) tested the effect of dipyridamole plus PALA (N-phosphonacetyl-L-aspartate), an inhibitor of *de novo* pyrimidine synthesis, and showed that dipyridamole substantially inhibited uridine uptake by neoplastic human cells, and caused about a 4-fold increase in the cytotoxicity of PALA. Zhen *et al.* (1983) and Fischer *et al.* (1984) tested the effect of dipyridamole plus acivicin on the growth of hepatoma 3924A and VACO5 cells, respectively, and showed that the protections provided by the nucleosides were blocked by dipyridamole. Moreover, Cabral *et al.* (1984) showed that dipyridamole enhanced MTX accumulation by sarcoma 180 cells and diminished the efflux of the drug. Nelson & Drake (1984) also tested the effect of this combination and showed that dipyridamole enhanced the toxicity of MTX on cells in culture and in mice.

On the contrary, dipyridamole reduced cytarabine uptake by normal mouse cells, and by L1210 murine leukaemia and HL-60 human leukaemia cells (King *et al.*, 1984).

We tested the effect of dipyridamole plus VCR for the first time in the present study. We found that these compounds had synergistic inhibitory effects on *in vitro* growth of cultured cells and that dipyridamole enhanced VCR accumulation in malignant haematologic cells. Dipyridamole also enhanced VCR accumulation in normal lymphocytes, but the efflux of VCR from normal lymphocytes was more rapid than that from malignant cells. Cytochalasin B, which enhances the accumulations of VCR and daunomycin in tumour cells, inhibits actin polymerization and binds to the cell membrane. Verapamil, a calcium channel blocker, also strongly inhibits outward transport of VCR. The mechanism of action of verapamil seems to be different from that of cytochalasin (Tsuruo & Iida, 1986). Dipyridamole seems to block entry of nucleosides and nucleoside analogues by binding tightly to the plasma membrane (Kessel & Dodd, 1972; Paterson *et al.*, 1980), although its exact mechanism of action is unknown. Further information on the mechanisms of membrane transport of drugs should be helpful in developing more effective methods of drug administration.

Dipyridamole is a vasodilator and antithrombotic agent, which has been used in the treatment of angina pectoris, while VCR is one of the most widely used anti-cancer drugs. Our results suggest that VCR should be more effective in the treatment of leukaemia and lymphoma when administered in combination with dipyridamole.

### References

BERLIN, R.D. & OLIVER, J.M. (1975). Membrane transport of purine and pyrimidine bases and nucleosides in animal cells. *Int. Rev. Cytol.*, **42**, 287.

CABRAL, S., LEIS, S., BOUER, L. NEMBROT, M. & MORDOH, J. (1984). Dipyridamole inhibits reversion by thymidine of methotrexate effect and increases drug uptake in sarcoma 180 cells. *Proc. Natl Acad. Sci. USA*, **81**, 3200.

EMMONS, P.R., HARRISON, M.J.G., HONOUR, A.J. & MITCHELL, J.R.A. (1965). Effect of a pyrimidopyrimidine derivative on thrombus formation in the rabbit. *Nature*, **208**, 255.

FARMER, J.L. & PRAGER, M.D. (1981). Inhibition of lymphoproliferation by dipyridamole. *Biochem. Pharmacol.*, **31**, 1381.

FISCHER, P.H., PAMUKCU, R., BITTER, G. & WILLSON, J.K.V. (1984). Enhancement of the sensitivity of human colon cancer cells to growth inhibition by acivicin achieved through inhibition of nucleic acid precursor salvage by dipyridamole. *Cancer Res.*, **44**, 3355.

HARKER, L.A. & KADATZ, R.A. (1983). Mechanism of action of dipyridamole. *Thrombosis Res.*, Suppl. IV, 39.

HIROSE, M., TAKEDA, E., NINOMIYA, T., KURODA, Y. & MIYAO, M. (1986). Inhibitory effect of dipyridamole on the growth of various human hematologic malignant cell lines. *Tokushima J. Exp. Med.*, **33**, 51.

HOWELL, S.B., MANSFIELD, S.J. & TAETLE, R. (1981). Thymidine and hypoxanthine requirements of normal and malignant human cells for protection against methotrexate cytotoxicity. *Cancer Res.*, **41**, 945.

KESSEL, D. & DODD, D.C. (1972). Effects of persantin on several transport systems of murine leukemias. *Biochem. Biophys. Acta.*, **288**, 190.

KING, M.E. & HOWELL, S.B. (1982). Inhibition of uridine uptake and potentiation of PALA cytotoxicity by dipyridamole. *Proc. Am. Assoc. Cancer Res.*, **23**, 207 (*abstract*).

KING, M.E., NAPORN, A., YOUNG, B. & HOWELL, S.B. (1984). Modulation of cytarabine uptake and toxicity by dipyridamole. *Cancer Treatment Rep.*, **68**, 361.

MINOWADA, J., OHNUMA, T. & MOORE, G.E. (1972). Rosette-forming human lymphoid cell lines. I. Establishment and evidence for origin of thymus-derived lymphocytes. *J. Natl Cancer Inst.*, **49**, 891.

MINOWADA, J. (1981). Marker profiles of leukemia-lymphoma cell lines. In *Modern Trends in Human Leukemia IV*, Neth, R. *et al.* (eds) p. 323. Springer Verlag: Berlin.

NELSON, J.A. & DRAKE, S. (1984). Potentiation of methotrexate toxicity by dipyridamole. *Cancer Res.*, **44**, 2493.

PABST, H.W. (1959). Über die wirkung einer neuen coronarer-weiterndern substanz. *Med. Klin.*, **7**, 257.

PATERSON, A.R.P., LAU, E.Y. & DAHLIG, E.A. (1980). A common basis for inhibition of nucleoside transport by dipyridamole and nitrobenzylthiosine? *Mol. Pharmacol.*, **18**, 40.

PAZDUR, J., KRZYSTYNIAK, K. & KOPEC, M. (1980). Inhibition of lymphoproliferation by dipyridamole. *Biochem. Pharmacol.*, **29**, 2515.

PINEDO, H.M., ZAHARKO, D.S., BULL, J.M. & CHABNER, B.A. (1976). The reversal of methotrexate cytotoxicity to mouse bone marrow cells by leucovorin and nucleosides. *Cancer Res.*, **36**, 4418.

SCHOLTISSEK, C. (1968). Studies on the uptake of nucleic acid precursors into cells in tissue culture. *Biochem. Biophys. Acta*, **158**, 435.

STEEL, G.G. & PECKHAM, M.J. (1979). Exploitable mechanism in combined radiotherapy-chemotherapy: The concept of additivity. *Int. J. Radiat. Oncol.*, **5**, 85.

TAKEDA, E., HIROSE, M., KURODA, Y. & 6 others (1984). Ribonucleotide reductase and thymidine kinase activities in various cultured cell lines derived from hematologic malignancies. *Gann*, **75**, 816.

TSURURO, T. & IIDA, H. (1986). Effects of cytochalasins and colchicine on the accumulation and retention of daunomycin and vincristine in drug resistant tumor cells. *Biochem. Pharmacol.*, **35**, 1087.

ZHEN, Y.S., LUI, M.S. & WEBER, G. (1983). Effects of acivicin and dipyridamole on hepatoma 3924A cells. *Cancer Res.*, **43**, 1616.